# Editorial Notes

**Published:** 1903-09

**Authors:** 


### Editorial Notes.

**British Medical Association.** — The 1903 Meeting of the British Medical Association at Swansea was smaller in numbers than many of those of previous years, but was none the less a very successful one. It will be remembered as the first meeting under the new Rules of the Association, in accordance with which there were several innovations, the most important being the transference of a part of the business of the Association to a body of Representatives elected by the Branches. There can be no doubt that it is a great advantage to the Association to have its business matters carefully debated by a large body of elected delegates, and not to be liable to have important matters decided haphazard by a snatch vote in an almost empty room under the misnomer of a General Meeting.

The way in which the Representatives' Meeting was conducted must be considered highly successful, and certainly has so far justified the anticipations of the reformers. A good deal of time was necessarily occupied at a first meeting in settling the rules of procedure for this and future meetings, but after this several matters of general importance were considered. The newly constituted Medico-Political and Ethical Committees were directed to inquire into the conditions of contract practice, and it is to be hoped that some practical method of dealing with this matter will be the outcome; the support of the Association was rightly promised to the Irish Poor Law Doctors in their struggle for a "living wage"; and further, a proposal, the issue of which is more doubtful, to form a Committee for Scotland was accepted. The meeting was thoroughly business-like, and whilst the matters brought before it were adequately discussed, those members who came prepared to give a display of their oratorical powers soon found that unless they kept to the point their efforts were in vain. Too great credit for the success of this meeting can hardly be given to the Chairman, Sir Victor Horsley; that so much business was got through so well is chiefly due to his able management of the meeting. It may be well to say that the Representatives *were* representative of the Branches, and comprised many well-known members of the Association, amongst others the President-elect, Dr. W. Collier.

The sections were very fairly attended, but no doubt the numerous excursions provided and the delightful surroundings of Swansea furnished many members with an excuse for a not too exhausting devotion to the scientific side of the meeting. The rooms in which the sections were held were not large, but were conveniently grouped together. The Pathological Museum deserves more than the passing word of notice which we are only able to give it. Though moderate in size, a most interesting set of specimens, chiefly macroscopical, had been collected; these were capitally arranged, so that what was in the museum could really be seen and studied—which is by no means always the case in these annual shows—and the general arrangements reflected great credit on the organisers, who are to be congratulated on the result of their by no means light labours.

As said above, Swansea lies in the midst of very beautiful surroundings, which are easily accessible by train, tram and water. Though ugly in itself, the town has a fine situation on the shore of its beautiful bay. For its size the town is singularly deficient in good buildings—there is hardly one of any consideration; this is a serious defect in entertaining such a large body as the British Medical Association, and the local committees deserve all the more credit for the success of their arrangements. The entertainments and excursions were well arranged, and so far as one could judge much enjoyed by the members present, who took full advantage of them. The feature that will perhaps most linger in the memory is that one could be on the seaside by a beautiful stretch of sands and within easy access of lovely coast scenery whilst still within the confines of a grimy and not attractive commercial city. We think that the members who were at the meeting will look back to it with pleasure, and with gratitude to the genial president and to the local committee for the very agreeable week which they owe to their efforts.

**The Medical Defence Union.**—The Annual Report for 1902 shows that the increase of membership has beaten any previous record. The medical profession appears to have now realised the importance of practically insuring against attacks which very often are made upon its members by unscrupulous persons, and the value of being defended in such attacks by an impersonal association is very fully acknowledged. The number of members on the register is 5,549, of whom 896 have been added during the year. It appears to be the duty of every member of the profession to join such a union as this, as no one of us can say how soon its assistance may be urgently needed, and the British Medical Association does not as yet seem prepared to undertake so large and important a responsibility.

**Bovine Tuberculosis.**—The questions relating to this subject are still much discussed, and further evidence is desired. The Royal Commission on Tuberculosis is collecting statistics, and has asked Dr. Odery Symes to collect evidence from this district. He will be glad to receive particulars of any case of abdominal tubercle verified by *post-mortem* examination, and attend or, if necessary, perform such examination. Dr. Symes is also desirous of obtaining information bearing upon the subject of the transmission of tubercle by means of food, and the relation of certain employments to tuberculosis.

